# Sternal reconstruction after post-sternotomy mediastinitis

**DOI:** 10.1186/s13019-017-0656-7

**Published:** 2017-11-02

**Authors:** Pankaj Kaul

**Affiliations:** Leeds General Infirmary, Great George Street, Leeds, LS1 3EX UK

**Keywords:** Deep sternal wound infections, Mediastinitis, Sternal dehiscence, Negative pressure wound therapy, Pectoralis major flaps, Rectus abdominis flaps, Omental grafts, Latissimus dorsi flaps, Free flaps, Allogeneic bone grafts

## Abstract

**Background:**

Deep sternal wound complications are uncommon after cardiac surgery. They comprise sternal dehiscence, deep sternal wound infections and mediastinitis, which will be treated as varying expressions of a singular pathology for reasons explained in the text.

**Methodology and review:**

This article reviews the definition, prevalence, risk factors, prevention, diagnosis, microbiology and management of deep sternal wound infections and mediastinitis after cardiac surgery. The role of negative pressure wound therapy and initial and delayed surgical management is discussed with special emphasis on plastic techniques with muscle and omental flaps. Recent advances in reconstructive surgery are presented.

**Conclusions:**

Deep sternal wound complications no longer spell debilitating morbidity and high mortality. Better understanding of risk factors that predispose to deep sternal wound complications and general improvement in theatre protocols for asepsis have dramatically reduced the incidence of deep sternal wound complications. Negative pressure wound therapy and appropriately timed and staged muscle or omental flap reconstruction have transformed the outcomes once these complications occur.

## Background

Deep sternal wound complications are uncommon after cardiac surgery. Deep sternal wound infections (DSWIs) are invariably accompanied by varying degrees of sternal dehiscence and mediastinitis. Alternately, even a purely mechanical sternal dehiscence will quickly get secondarily infected unless rewiring is undertaken expeditiously. In this article, therefore, deep sternal wound complications will be presumed to include deep sternal wound infections (DSWIs), sternal dehiscence (SD) and mediastinitis, and will be treated as a singular entity which might have varied expression rather than qualitatively different pathologies. The author recognises that mediastinits and deep sternal wound infections after cardiac surgery can occur without frank sternal dehiscence, but what invariably perpetuates a deep sternal wound infection is a communication of presternal tissues with mediastinum.

Deep sternal wound infections, sternal dehiscence and mediastinitis have moved from being fearsome complications of cardiac surgery to manageable problems. Negative pressure wound therapy has revolutionised the ward-based management of deep sternal wound infections. Pectoralis, rectus and latissimus flaps and omental grafts are commonest forms of sternal reconstruction although free flaps, intercostal perforator flaps, breast flaps and allogeneic bone grafts have been used.

## Methodology

A literature search was done using Healthcare Databases Advance Search comprising in particular PubMed and Medline databases up to June 2017 using MeSH headings: Sternal Wound Infections, Sternal Dehiscence, Sternal Reconstruction, Latissimus Dorsi Flaps, Omental Flaps, Rectus Abdominis Flaps, Pectoral Flaps, Negative Pressure Wound Therapy.

## Review

We present a review of prevalence, risk factors, prevention, diagnosis, microbiology and pathogenesis of sternal wound infections and mediastinitis. We discuss management of early and later, and therefore mostly infected, dehiscences and the central contribution of negative pressure wound therapy. The crucial role of various muscle flaps including pectoralis, rectus and latissimus flaps and omental grafts is specifically elaborated upon. Also presented is a review of some newer approaches that have been recently introduced to manage this complication that can prove particularly devastating if not treated early and well.

### Prevalence

Deep sternal wound complications occur in 0.8% to 1.5% patients and as high as 8% when bilateral IMAs are used [1, 2]. Cheung et al. reported on the variability of the impact of postoperative mediastinitis on survival after valve operations with mortality ranging from 6% to 70% [3]. Lazar reports 1% to 4% incidence of deep sternal wound infections in all cardiac procedures and describes the increased morbidity and mortality and decreased long-term life expectancy associated with mediastinitis [4]. There is general consensus that early treatment reduces mortality.

### Risk factors

Risk factors for deep sternal wound complications include breaches in theatre asepsis, long operations [5], postoperative bleeding with reexploration [6] or undrained retrosternal hematoma [2], poor wound closure [7] including use of excessive rewiring materials or techniques which strangulate tissues like Robiscek closure [8] or sternal plates [9], obesity [5], older age, previous CABG and 3 or 4 NYHA functional class [10], diabetes [11], COPD [4], hospital acquired pneumonias [12], dialysis [13], prolonged mechanical ventilation [14], etc. Looking at major sternal wound infections after open heart surgery, Ottino et al. did a multivariate analysis of 2579 consecutive open heart operations and found hospital environment, interval between admission and surgery, reoperation, blood transfusions, early chest reexplorations and sternal rewiring as significant variables on multiple stepwise logistic regression [6]. Tiveron et al. identified use of preoperative intraaortic balloon pump, haemodialysis and preoperative extracardiac vascular intervention to increase the chance of mediastinitis 4.4 to 5.4 times higher after multivariate analysis of 2768 patients who underwent cardiac surgery out of which 35 (1.5%) developed mediastinitis [15]. Weinstein et al. sought to identify risk factors of infected versus sterile wound dehiscence and, on multivariate regression, found BMI > 30, cardiac ejection fraction <40% and respiratory failure to be significant positive predictors for SSD and IMA grafting and respiratory failure significant positive predictors for SWI [16].

### Prevention

Prevention of sternal wound complications should be the cornerstone of all cardiac surgery. A precise aseptic technique, reducing personnel traffic in theatres, shorter and less complex procedures and heightened awareness of asepsis in theatre and ICU all reduce the prevalence of infection [17]. Prophylactic antibiotic administration before skin incision and another dose either before the sternal closure or within the second half life of the antibiotic is generally recommended [18, 19]. Use of topical vancomycin thrombin powdered gelatin paste [20], topical collagen-gentamicin sponges [21] or implants [22] has been reported to significantly reduce sternal wound infection but this is based on as few as 3 randomised controlled trials [20–22]. Harvesting internal mammary arteries without the surrounding retrochondral tissues including pleura, intercostal muscle and endothoracic fascia thus preserving the collateral blood supply to the sternum by the so called skeletonised technique in contradistinction to the more conventional pedicled technique in unilateral harvests and more importantly in bilateral IMA grafting in the special context of diabetics was acknowledged by the STS guidelines on arterial conduits for CABG [23]. Removal of intravascular and urinary catheters and pacing wires expeditiously, early extubation and ambulation, early discharge from ICU and hospital all generally help towards normal wound recovery [4, 17]. Procedural technicalities including use of sternal bands at 3rd intercostal space in addition to standard osteosynthesis with 8 wire cerclages [24] and application of rigid plate fixation [25] and titanium sternal plating with prophylactic NPWT have been variously and anecdotally reported to prevent postoperative mediastinitis. Lazar et al. summarised the recommendations for prevention of wound infections in an excellent paper: nasal swabs or PCR testing, intranasal mupirocin within 24 h of surgery and to be continued for 5 days in the absence of a negative swab or negative PCR testing for staphylococci, chlorhexidine bath or shower on the evening before surgery, cephalosporin antibiotic within 60 mins of surgery, repeat if procedure longer than 4 h and not more than 48 h, vancomycin in patients with allergic to beta lactams or when MRSA is a special concern, addition of aminoglycoside to vancomycin for 1 preoperative dose, enteral nutrition for 7–10 days in patients with preoperative hypoalbuminaemia, treatment of all distant extrathoracic infections before surgery, smoking cessation and aggressive pulmonary toilet, continuous insulin infusion if glucose levels > 200 mg/dL to keep blood sugar levels below 180 mg/dL for first 24 h, topical antibiotics to sternal edge, no bone wax, figure-of-eight sternal closure, Robicsek weave technique in multiple fractures, sternal plates or bands in high-risk patients, external chest support vests, good haemostasis, early extubation and early removal of indwelling urinary and central venous catheters [4].

### Diagnosis

Diagnosis of deep sternal wound infection, dehiscence or mediastinitis is generally made clinically. Disproportionate pain on coughing, persistent wound discharge, appreciation of movement of two sternal halves relative to each other unmasked by deep inspiration or coughing are the usual warning signs. Indeterminate malaise, fever, rising WBC count and CRP, deteriorating renal function, widened mediastinum and displaced sternal wires are occasionally seen. CT scan showing sternal disruption and retrosternal fluid with air pockets is diagnostic although some edema and clot is present in mediastinum routinely after cardiac operations [26].

### Microbiology and pathogenesis

Gardlund et al. identified three different types of postoperative mediastinitis in cardiac surgery with respect to microbiology and pathogenesis: 1. mediastinitis associated with obesity, chronic obstructive airway disease and sternal dehiscence, typically caused by coagulase negative staphylococci 2. mediastinitis following peroperative contamination of mediastinal space, often caused by staph aureus 3. Mediastinitis mainly caused by spread from concomitant infection in other sites during postoperative period, often caused by gram negative rods [27]. A large number of DSWIs are caused by staphylococcus species and many of these infections arise from patient’s own nasal flora. The risk of S aureus infection is increased three-fold in carriers and 20% to 30% of general population are carriers of S aureus. The risk of postoperative MRSA bacteraemia is higher in MRSA carriers than risk of postoperative MSSA bacteraemia in MSSA carriers whilst 5% to 15% of all ICU admissions are MRSA carriers [4].

### Treatment of wound infections

The principles of the treatment of wound infections are standard and involve drainage of infected spaces, debridement of necrotic tissue, antibiotic therapy and strategies to achieve closure of sternal space. Quite often the treatment has to be individualised based on the infecting organism, patient’s status and, most crucially, the depth of infection [28]. Immediate closure can be performed after debridement if there is enough sternum to rewire and achieve mediastinal stability. If there is significant residual mediastinal infection, this can be combined with negative pressure wound therapy (NPWT) rather than mediastinal irrigation with dilute povidone iodine which has been reported to be systemically absorbed on occasions resulting in diverse complications including renal failure, electrolyte imbalances, changes in iodine metabolism, abnormal thyroid function, mental changes and seizures [29, 30]. On occasion when rewiring is possible but soft tissue closure over the rewired sternum is not possible, a pectoral advancement or flap can be added to the rewiring. When there is substantial loss of sternal bone and rewiring is not possible, a muscle flap operation needs to be done. If the loss of bone is mainly in the manubrium or upper half of the body of sternum, bilateral pectoral flaps suffice. If the loss, however, is mainly in the xiphisternum or the lower body of the sternum or involves xiphisternum and the lower body in addition to the upper sternum, bilateral pectoral flaps may not be able to cover the lower part of the wound unless the insertion of one or both pectorals is divided in the deltopectoral groove. Rarely this does not suffice and addition of usually the right rectus major muscle based on superior epigastric artery pedicle needs to be considered. If the loss is particularly in the lower sternum and manubrium is relatively spared, greater omental graft based on right gastroepiploic artery and rarely left gastroepiploic artery is quite suitable although greater omentum often is substantial enough to extend all the way to the suprasternal space. In massive necrosis of sternum and soft tissues and skin, once infection has been brought under control by NPWT, the only option may be a latissimus dorsi myocutaneous flap requiring a posterolateral incision and rotation of the flap anteriorly. Some patients, however, may not be candidates for immediate flap closure because of severe mediastinitis and insufficiently drained infective mediastinal spaces. Negative pressure wound therapy has been used as a bridge to flap or omental closure in these patients.

### Negative pressure wound therapy

Negative pressure wound therapy (NPWT) applies subatmospheric pressure to the surface of wound, removes excessive fluid, decreases wound edema, accelerates wound healing and granulation tissue formation, stabilises chest wall, increases sternal blood flow and has been shown to improve early and long term survival in patients with deep sternal wound infection. It has emerged as one of the most significant developments in the last two decades in the management of sternal wound infections [31, 32]. All deep sternal wound infections and all limited sternal wound dehiscences not resulting in frank sternal instability should be treated in the first instance with negative pressure wound therapy and antibiotics. A significant number will heal satisfactorily with secondary intention with no requirement for further reconstructive management. This therefore represents a triumph for this vacuum assisted negative pressure management of wounds and a departure from the previous norm of either immediate return to theatre or weeks and rarely months of debilitating wet wound dressings sometimes a number of times the same day. Looking at the role of NPWT in cardiac surgical wound complications, Moriasky et al., in a propensity score matching analysis, reported significantly reduced in-hospital mortality in 22 patients treated with NPWT compared to 22 who did not (5 vs 36%, *P* = 0.021). In DSWI infected with MRSA, in particular, NPWT significantly reduced in-hospital mortality caused by DSWI (0 vs 52%, *P* = 0.003). Equally importantly, NPWT as a bridge therapy to tissue flaps may play a major role in treating DSWI and improve prognosis for patients with MRSA-infected sternal wounds [31]. Debreceni et al. reported on 62 consecutive patients who underwent vacuum assisted wound closure (VAC). 28 had immediate or delayed wound resuture and 34 had subsequent well- vascularised pectoral muscle and/or omental or pericardial fat pad flaps with 11.3% in-hospital mortality and only 3.6% recurrence rates [32]. Fuchs et al. compared NPWT with drainage, conventional dressings and flap closure and reported reduced time to achieve negative cultures, reduced hospital stay and lower percentage of patients discharged with an open sternum. Raja et al. reported that NPWT reduced the time interval between sternal debridement and primary or flap closure of infected sternal wounds.

### Early dehiscence

All sternal dehiscences, whether sterile or infected, causing frank sternal instability result in considerable pain, respiratory distress and occasionally haemodynamic compromise and need to return to theatre for immediate reoperation. This involves removal of all sternal wires, suture and necrotic material. If sternum is generally healthy and well vascularised, rewiring after limited sternal debridement suffices. There is no substitute to native healthy sternum in front of heart. The author is not a great believer in continuous warm saline, povidone-iodine or antibiotic irrigation postoperatively. Their benefit is doubtful [33] but there is a real risk of tamponade if all infused fluid is not effectively evacuated. If there is insufficient muscular tissue and fat to close in front of rewired sternum, limited myocutaneous advancement pectoral flaps will be required. Redivac suction drains left for a few days under the muscle flaps will usually prevent seroma formation.

### Infected sternal dehiscences – An overview

A real challenge in sternotomy wound complications comes from patients who develop early infected sternal dehiscences with substantial loss of sternal bone with considerable mediastinal instability and relative movement of the residual sternal plates. This is obviously not amenable to rewiring and despite filling the residual space with muscle flap there might still be residual relative movement of sternal plates with mediastinal instability. Sternal debridement followed by NPWT till mediastinal stability is achieved by tissue adhesions followed by reconstructive flaps is probably preferable in these patients but careful and close observation is required.

Most patients with infected sternotomy wounds with loss of sternal bone or soft tissue will require reconstructive surgery requiring mostly muscle flaps individually or in combination, preferably after a few weeks following initial surgery, allowing for mediastinal adhesions and stability. The most commonly used flaps are pedicled pectoralis major, rectus abdominis and omental flaps. Less commonly pedicled latissimus dorsi flaps may be used. Rarely free flaps with AV loops may be used. Anecdotally, internal mammary artery perforator (IMAP), superior epigastric artery perforator (SEAP), intercostal artery perforator (ICAP) flaps, breast flaps, falciform ligament flaps or free gracilis flaps have been employed. Bilateral pectoralis major flaps based on thoracoacromial artery will cover most of the sternal wound except occasionally the lower 1/4th. The sternal and costal origins of the muscle are dissected off the bone and clavicular origin left intact. If the insertion of the pectoralis major tendon to the anterior lip of the humeral intertubercular sulcus is divided in the deltopectoral groove, the muscle can be mobilised inferomedially to reach the lowest part of the sternal wound. Pectoralis major flaps are the workhorse flaps for sternal reconstruction and can reach almost all sternal wounds. Apart from occasional seromas and rare incidences of shoulder weakness when the insertion is divided, they are relatively free of side effects. Pedicled rectus abdominis flaps are especially suited to the lower 1/4th of the sternal wound and are often used in combination with the pectoral flaps. They obviously need a long abdominal incision unless harvested laparoscopically. They are based on the superior epigastric artery and therefore a contralateral flap is used when LIMA has been used although there is evidence of reasonable collateral supply even when a flap ipsilateral to IMA harvest is used. Rectus abdominis flap is easy to harvest and is a turn over flap not requiring considerable tissue dissection but may lack muscle mass to support extensive sternal loss. Omental flaps based on right gastroepiploic artery and unusually on left gastroepiploic artery provide more vascular tissue potentially with greater infection neutralising properties and have been used extensively. There have been anecdotal reports of their use when there is obvious infection of the heart and great vessels. Their obvious disadvantages include the need to enter peritoneal cavity in the presence of mediastinitis and rare herniations. They however can be harvested laparoscopically. Latissimus dorsi flaps provide extensive bulk and can be employed as both muscle and myocutaneous flaps and are uniquely suited when there is massive sternal loss due to necrosis. They clearly need large harvesting incisions but do not disrupt blood supply to sternum and parasternal tissues.

### Pectoralis major flaps

Wu et al. reported 19 patients who underwent bilateral pectoralis major flaps for deep sternal wound infections (DSWI) out of which 4 needed additional rectus abdominis (RA) flap because the pectorals would not reach the inferior part of the wound. No patient showed infection recurrence [34]. Preminger et al. reported on 25 out of 174 wounds which underwent limited sternal debridement and partial myocutaneous advancement flaps which they reported to be as effective as muscle flaps but less invasive with less operative time, blood loss and length of hospitalisation [35]. Muscle flaps require posterior mobilisation of the pectoral muscle from its sternal and costal attachments as well as anterior mobilisation from the subcutaneous tisssues. Myocutanoeous advancement flaps, on the other hand, require only posterior dissection from the sternum and costal attachments. Eriksson et al. discussed functional impairment after pectoral flaps in the long term with an average follow up of 5.9 years. They found significant longterm disability in one third of patients. Sternum had not been closed in half the patients after debridement and the fact that the function of right arm and shoulder was affected more often despite the majority of procedures being left sided suggests that loss of skeletal continuity of the chest wall was more disabling than loss of pectoral muscle function [36]. Longstanding sternal non-union of 5 to 48 months was treated by pectoralis major muscle flap reconstruction in 24 patients over a 15 years period by Cabbabe and colleagues who reported dramatic improvement in symptoms of pain, popping and grinding [37]. Tomos et al. emphasised the preservation of the perforating branches of the non-grafted IMA along with the thoracoacromial vessels as well as non-detachment of humeral attachment by a second cutaneous incision as a simpler and equally effective alternative technique [38]. In a large series of 114 consecutive patients, Ascherman et al. recommended one stage debridement and immediate closure with bilateral pectoralis major myocutaneous advancement flaps with 7.9% 30 days mortality and 16.7% postoperative morbidity including partial dehiscences, skin edge necrosis and seromas [39]. Sung et al. confirmed normal development and no limitations to upper trunk or limb movements in 11 patients with advanced pectoral major flaps with DSWIs [40]. Sung et al. reported bilateral pectoralis and rectus abdominis triple muscle flap in a 2 months old patient who had arterial switch surgery complicated by Enterobacter mediastinitis requiring multiple debridements earlier. Further surgery for pulmonary stenosis through reconstructed sternum was uneventful [41]. Dosios extended the use of pectoralis major and rectus abdominis myocutaneous flaps to chest wall tumours, radionecrosis of chest wall, bronchopleural fistulae in addition to deep or chronic sternal wound infections with good results [42]. Backer et al. used, in 8 children, combinations of pectoralis major, rectus and cervical strap muscle vascularised flaps in a variety of indications including exposed homograft, exposed Gore-tex graft, exposed right ventricle, orthotopic heart transplants with sternums left open and a Marfan patient with Pectus and false aortic aneurysm [43]. Ascherman et al. described single stage pectoralis major myocutaneous advancement flaps in 20 consecutive heart transplant recepients with 5% wound infection and 20% seromas and excellent functional and aesthetic results [44]. Li emphasised the harvesting of the entire pectoralis major muscle to facilitate the closure of the lower portion of sternal wound resulting in far less frequent need of abdominal flaps and the morbidity associated with them. Out of 69 patients, 90% received pectoralis major flaps and 58% received only one pectoralis major flap [45].

### Rectus abdominis flaps

Kuntscher et al. reported on the versatility of 7 superiorly based vertical musculocutaneous rectus abdominis flaps for reconstruction of osteocutaneous defects following sternal osteomyelitis and tumour resection, with occasional requirement of arterial and/or venous recharging, with no flap loss [46]. Netscher et al. described 5 patients in whom either both IMAs were used for coronary revascularisation or in whom there was a contralateral subcostal incision mandating use of at least one rectus abdominis muscle flap ipsilateral to LIMA ligation. All 5 flaps were successful and an injection study revealed rich collateral circulation to the superior epigastric vascular pedicle through the musculophrenic and lower intercostal arteries. They suggested that one could reliably maintain a viable rectus muscle flap even in the face of ipsilateral IMA ligation if elevation of the rectus muscle and division of the lateral segmental vessels is done only till the costal margin [47]. Davison et al. in a retrospective study based on 130 patients, including 41 bilateral pectoralis major flaps and 56 rectus flaps, concluded that rectus abdominis alone was superior to the coverage of inferior sternum compared to pectoral flaps with rectus fascia extensions. They opined that sternal wounds should be covered preferentially by a pectoralis flap to cover superior infection and by a rectus flap if the dehiscence is localised to the distal third [48].

### Omental grafts

Lovich et al. described their experience of 20 patients with postcardiotomy sternal wound infections 17 of whom underwent omental pedicle grafts with excellent results. A left subcostal incision began to be used for omental harvesting to avoid incisional hernia towards the later part of their practice [49]. Brabendare et al. reported good functional and aesthetic results in 6 consecutive patients with laparoscopically harvested omentoplasty in combination with prior negative wound cultures achieved with repeated wound debridements, antibiotics and negative pressure wound therapy (NPWT), supplemented by bilateral pectoralis major advancement flaps [50]. Acarturk emphasised the large size and bulk of omental flaps to fill 3-dimensional dead spaces and the advantages of their large pedicles and rich vascular and lymphatic networks. Out of 9 laparoscopic omental harvests, 7 were used for reconstruction of infection related sternal wound complications and 2 for repair of intrathoracic viscera with excellent results, apart from one transdaiphragmatic hernia treated laparoscopically [51]. Levites et al. described sternal wound reconstruction with falciform and omental flaps for chronic sternal osteomyelitis (42A). Salameh et al. treated 7 complex mediastinal wounds with omental flaps based on right gastroepiploic artey [3], left gastroepiploic artery [1] or both [3] along with 5 bilateral pectoralis major myocutaneous advancement flaps with good results [52]. Schroeyers et al. after disappointing results with conservative management of post-CABG mediastinitis, treated 32 patients with combination of only omentoplasty (*n* = 11), a single pectoralis major flap and omentoplasty (*n* = 20) and bilateral pectoral flaps only (n = 1) with no early or late flap failures [53]. Milano et al. compared 21 isolated omental flaps and 38 pectoral flaps for poststernotomy mediastinits and found omental flaps had improved early outcomes with no late flap failures and were more effective therapy relative to pectoral flaps [54]. Parissis et al. reported on the outcomes and risk analysis of DSWIs with specific emphasis on omental transposition in a large series of 3896 cardiac surgical patients with 120 wound infections (3.02%) out of which 52 (1.34%) had DSWIs. Diabetes, renal dysfunction and prolonged mechanical ventilation were the commonest risk factors for DSWIs. Overall mortality was 9.3% and the mortality for the omental group was 8.3% with a mean hospital stay of 59 days [55]. Pieri et al. reported on a higher incidence of medistinitis in patients who underwent implanatable ventricular assist devices (VAD) and the role of well-vascularised and immune active omental flaps after aggressive debridement and NPWT in this selective group. There was a 47% survival, with postoperative renal failure requiring dialysis and septic shock requiring vasopressors the greatest risk factors for death [56]. Dorseifer et al. looked beyond the pedicled local and regional grafts and arteriovenous microsurgical loops and reported 12 patients with DSWIs who underwent gracilis (*n* = 8) and anterolateral thigh perforator free flaps (*n* = 4) anastomosed to the right gastroepiploic vessels harvested in 42% patients laparoscopically with uneventful healing [57]. Kaul et al. described a chronic encapsulated anterior mediastinal abscess presenting with remote cutaneous fistulisation 12 years after redo aortic valve replacement for prosthetic valve endocarditis. The abscess cavity was excised and the thin posterior rim of the cavity which formed the anterior wall of ascending aorta was covered with right gastroepiploic artery based pedicled omental flap [58]. Omental flaps may be the seat of secondaries from a primary cancer and Telfer et al. reported metastatic colonic adenocarcinoma in a pedicled omental flap used for sternal reconstruction [59].

### Latissimus dorsi flaps

Meland et al. described intrathoracic transposition of extrathoracic skeletal muscle in intrathoracic infections associated with infection, leakage or bleeding of airway, lung parenchyma, esophagus, heart or great vessels [60]. Tizian et al. described 5 patients with total sternal necrosis with exposure of anterior part of heart and ascending aorta including prosthetic grafts who underwent latissimus dorsi muscle flap transposition [61]. Hakala et al. reported repair of a full-thickness right ventricular defect with a de-epithelialized myocutaneous latissimus dorsi flap following failure of a PTFE patch repair of a rupture of right ventricle following sternal wound infection [62]. Kaul described latissimus dorsi myocutaneous flap reconstruction due to sternal non-union following 5 previous sternotomies in the same patient for repeated successful surgical rescues of early and delayed multiple ruptures of ventricular septum, right ventricle and aneurysmal left ventricle following massive biventricular infarction [63]. Latissimus dorsi muscle flaps preserve collateral blood supply to the sternum and parasternal tissues with a short harvest time although the flap mobilisation has to be done in a lateral decubitus position and the patient turned over later [64].

### Free flaps and perforator flaps

Taeger et al. described 8 patients with extensive deep sternal osteomyelitis requiring total sternectomy where the large defect was reconstructed by free flap transplantation using the vastus lateralis myocutaneous flap, rectus abdominis and bipedicled latissimus dorsi/parascapular flap. As the local recipient vessels were depleted in all patients, the pedicle of the flap was anastomosed to a high-flow and short-length subclavian arteriovenous loop as recipient vessel in all cases [65]. Wang et al. described 15 patients who underwent excision of inflamed, infected and unstable lower sternal and abdominal keloids with sixth internal mammary artery perforator (IMAP) and superior epigastric artery perforator (SEAP) flaps followed by early postoperative low dose radiation therapy. The preliminary results were encouraging and perforator selection and flap design were based on preoperative multidetector-row computed tomographic angiography which correlated well with the intraoperative findings [66]. Bravo et al. too described 4 patients with free style local perforator flaps for sternal defects using audible Doppler signals to localise the perforators [67].

### Breast flaps

Cooper et al. described the use of inferomedial fasciocutaneous breast flaps to reconstruct sternal dehiscence with concomitant soft tissue loss [68]. De Fontaine combined reduction mammoplasty with pectoralis major flaps in patients with infected sternal wounds following cardiac surgery due to inferolateral tension on the wound due to macromastia [69].

### Allogeneic bone grafts

Pancholy et al. used sternal reconstruction with sternal plating and local use of bone morphogenetic protein (BMP) and demineralised bone matrix, preceded by NPWT and followed by pectoral flaps to achieve good sternal stability as seen at 3 and 6 months follow up [70]. Kalab et al. described transplantation of allogeneic bone graft of sternum (*n* = 10), calva (n = 1) and only crushed spongy bone (*n* = 2) along with transverse titanium plates and bipectoral flaps as a promising and easily applied method in serious sternal bony loss [71].

### Comparisons

There are a number of studies which either describe use of a combination of flaps or which offer comparative analyses of the various types of flaps for sternal reconstruction. Lindsey described his experience with 47 patients with DSWE undergoing reconstruction with pectoral and rectus flaps (62% had single right split medially –based pectoralis major flap) and observed striking differences between outcomes of flap reconstruction within or beyond 5 days of wound drainage and debridement, with 23% major wound complications in the first group when flap reconstruction was done within 5 days of dehiscence [72]. Describing his experience with 415 consecutive patients with omental and pectoral flaps, Izaddoost et al. warned about blanket single stage debridement and flap coverage of purulent wounds. An early mortality of 25% was reduced to 1.5% by a multi-staged, multiple flap approach employing staged debridements of non-viable tissue depending on the purulence and a variety of flaps depending on geographical severity of sternal loss with strict adherence to the general principles of early detection, debridement and elimination of dead space [73]. Van Wingeden et al. identified 333 citations and 16 observational studies covering 1046 patients representing the best evidence on the relative advantage of muscle flaps over omental flaps in the management of DSWIs. Weight of current evidence seemed to be insufficient to prove the superiority of reconstruction with muscle flaps to a laparotomy-harvested omental flap in the treatment of DSWIs. On the other hand, there was a slight but not significant survival advantage with omental flaps. Data on complications following flap closures, unadjusted for potentially confounding factors, showed a higher incidence of complete or partial flap loss, haematoma, arm or shoulder weakness and chronic chest wall pain as well as recurrent chest wall infection after muscle flaps [74]. Kaye et al. reported 136 sternal reconstructions over 7 years with right pectoral turnover, left pectoral advancement and omental flaps with 9.6% overall mortality. 50% heart transplant patients developed wound infections (all of them had diabetes and renal failure) and all 10 of them received omental transplants. 25 different organisms were identified and 18.5% had multiple organisms [75]. Spartalis et al. emphasised the usefulness of additional omental flaps to reconstruct the inferior parts of infected sternal wounds requiring reconstruction to supplement bipectoral musculofascial advancement flaps in 55% patients with DSWIs with 5.4% mortality [76].

## Need for plastic surgeon

All cardiac surgeons should be able to construct pectoralis major muscle flaps or myocutaneous advancement flaps which are the work-horse sternal reconstruction flaps. This is in the anatomical area of their usual site of activity and will address around 80% of all sternal reconstructions. The pectoralis major needs to be dissected off from the chest wall origin and that is all that needs to be done for the advancement flaps. For the pectoralis major muscle flap, the muscle in addition needs to be dissected from the overlying subcutaneous tissue and then stitched to the contralateral muscle in front of the rewired or debrided sternum. Only a small number of patients with extensive sternal defects will need a further rotation of the muscle inferomedially and this can be done easily by a separate incision in the dectopectoral groove and dividing the insertion of the pectoral head to the lateral lip of the bicipital groove of the humerus and anterior lip of deltoid tuberosity. It is important to preserve the pectoral branch of the thoracoacromial artery which is the main blood supply to the pectoralis major. When there is substantial sternal loss inferiorly, rectus abdominis flap might need to be constructed in addition to the pectoralis flaps. This is done by a long midline abdominal incision and dissecting off the muscle within the sheath based on the superior epigastric artery after dividing it inferiorly. Usually the side contralateral to the LIMA harvest is taken. This can also be done by a cardiac surgeon easily. Cardiothoracic surgeons who have reasonable experience of thoracic surgery should be well within their comfort zone to harvest latissimus dorsi myocutaneous flaps for anterior turn-over when encountering substantial sternal and soft tissue loss. The pedicle is based on thoracodorsal artery. All cardiothoracic surgeons who have had general surgical training will be able to harvest greater omentum based on right gastroepiploic artery and using it for reconstructions in inferior or total sternal problems. It is particularly important to pay attention to the greater curvature of the stomach and the middle colic artery in the transverse mesocolon.

In general, many cardiac surgeons will perform most of the above procedures with a plastic surgeon. However, I do feel that as cardiac surgeons we should endeavour to train ourselves to be able to treat all the site-specific wound complications that we encounter.

## Conclusions

Better understanding of risk factors that predispose to deep sternal wound complications and general improvement in theatre protocols for asepsis have dramatically reduced the incidence of deep sternal wound infections, sternal dehiscence and mediastinitis following cardiac surgery. Negative pressure wound therapy (NPWT) and appropriately timed and staged muscle or omental flap reconstruction have revolutionised the outcomes once these complications occur.

## Abbreviations

CRP: C reactive protein
DSWI: Deep sternal wound infection
ICAP: Intercostal artery perforator
IMA: Internal mammary artery
IMAP: Internal mammary artery perforator
LIMA: Left internal mammary artery
NPWT: Negative pressure wound therapy
PTFE: Polytetrafluoroethylene
SD: Sternal dehiscence
SEAP: Superior epigastric artery perforator
SSD: Superficial sternal dehiscence
VA: Vacuum assisted closure
WBC: White blood cell

## References

[1] Bryer RH, Mills SA, Hudspeth AS, Johnston FR, Cordell ARA (1984). Prospective study of sternal wound complications. Annals of thoracic. Surgery.

[2] Culliford AT, Cunningham JN, Zeff RH, Isom OW, Teiko P, Spencer FC (1976). Sternal and costochondral infections following open heart surgery. A review of 2594 cases. J Thorac Cardiovasc Surg.

[3] Cheung EH, Craver JM, Jones EL, Murphy DA, Hatcher CR, Guyton RA (1985). Mediastinitis after cardiac valve operations: impact upon survival. J Thorac Cardiovasc Surg.

[4] Lazar HL, Vander Salm T, Engelman R, Orgill D, Gordon S (2016). Prevention and management of sternal wound infection. J Thorac Cardiovasc Surg.

[5] Wilson AP, Livesey SA, Treasure T, Gruneberg RN, Sturridge MF (1987). Factors predisposing to wound infection in cardiac surgery: a prospective study of 517 patients. European Journal of Cardiothoracic Surgery.

[6] Ottino G, De Paulis R, Pansini S, Rocca G, Tallone MV (1987). Major sternal wound infection after open heart surgery: a multivariate analysis of risk factors in 2579 consecutive operative procedures. Annals of thoracic. Surgery.

[7] Sarr MG, Gott VL, Townsend TR (1984). Mediastinal infection after cardiac surgery. Annals of thoracic. Surgery.

[8] Schimmer C, Reents W, Berneder S, Eigel P, Sezer O (2008). Prevention of sternal dehiscence and infection in high risk patients: a prospective randomised multicentre trial. Annals of thoracic. Surgery.

[9] Grabert S, Erlebach M, Will A, Lange R, Voss B (2016). Unexpected results after sternal reconstruction with plates, cables and cannulated screws. Interact Cardiovasc Thorac Surg.

[10] Hassan M, Smith JM, Engel AM (2006). Predictors and outcomes of sternal wound complications in patients after coronary artery bypass grafting. Am Surg.

[11] Kouchoukos NT, Wareing TH, Murphy SF, Pelate C, Marshall WG (1990). Risks of bilateral internal mammary artery bypass grafting. Annals of thoracic. Surgery.

[12] Reyna GC, Baca GGA, Concebida LEM, Sanchez GB, Villegas GP, Sanchez RA (2006). Risk factors for mediastinitis and sternal dehiscence after cardiac surgery. Rev Esp Cardiol.

[13] Demmy TL, Park SB, Liebler GA, Buckholder JA, Maher TD, Benckart DH (1990). Recent experience with major sternal wound complications. Annals of thoracic. Surgery.

[14] Newman LS, Szezukowski LC, Bain RP, Perlino CA (1988). Suppurative mediastinitis after open heart surgery; a case control study of risk factors. Chest.

[15] Tiveron MG, Fiorelli AI, Mota EM, OSAV M (2012). Preoperative risk factors for mediastinitis after cardiac surgery: assessment of 2768 patients. Brazilian Journal of Cardiovascular Surgery.

[16] Weinstein AL, Chang MM, Argenziano M, Ascherman JA (2016). Risk factors of infected sternal wounds versus sterile wound dehiscence. J Surg Res.

[17] Ferrazi P, Allen R, Crupi G, Reyes I, Parenzan L, Maisonnet M (1986). Reduction of infection after cardiac surgery: a clinical trial. Annals of thoracic. Surgery.

[18] Myerowitz PD, Caswell K, Lindsay WG, Nicoloff DM (1977). Antibiotic prophylaxis for open heart surgery. J Thorac Cardiovasc Surg.

[19] Engelman R, Shahian D, Shemin R (2007). The Society of Thoracic Surgeons practice guideline series: antibiotic prophylaxis in cardiac surgery, part II: antibiotic choice. Annals of thoracic. Surgery.

[20] Vander Salm TJ, Okike ON, Pasque MK (1989). Reduction of sternal infection by application of topical vancomycin. J Thorac Cardiovasc Surg.

[21] Frieberg O, Svedieholm R, Soderquist B (2005). Local gentamicin reduces sternal wound infections after cardiac surgery: a randomised controlled trial. Annals of thoracic. Surgery.

[22] Eklund AM, Valtonen M, Werkkala KA (2005). Prophylaxis of sternal wound infections with gentamicin-collagen implant: a randomised controlled study in cardiac surgery. J Hosp Infect.

[23] Aldea GS, Bakaeen FG, Pal J (2016). The Society of Thoracic Surgeons clinical practice guidelines on arterial conduits for coronary artery bypass grafting. Annals of thoracic. Surgery.

[24] Reiss FC, Awwad N, Hoffmann B, Bader R, Helmold HYA (2004). Steel band in addition to 8 wire cerclages reduces the risk of sternal dehiscence after median sternotomy. The Heart Surgery Forum.

[25] Song DH, Lohman RF, Renucci JD, Jeevanandam V, Raman J (2004). Primary sternal plating in high risk patients prevents mediastinitis. European Journal of Cardiothoracic Surgery.

[26] Kay HR, Goodman LR, Teplick SK, Mundth ED (1983). Use of computed tomography to assess mediastinal complications after median sternotomy. Annals of thoracic. Surgery.

[27] Gardlind B, Bitkover CY, Vaage J (2002). Postoperative mediastinitis in cardiac surgery – microbiology and pathogenesis. European Journal of Cardiothoracic Surgery.

[28] Rupprecht L, Schmid C (2013). Deep sternal wound complications - an overview of old and new therapeutic options. Open J Cardiovasc Surg.

[29] Zec N, Donovan JW, Aufiero TX. Seizures in a patient treated with continuous povidone-iodine mediastinal irrigation. N Eng J Med 1992;326:1784. 10.1056/NEJM199206253262618 1301013

[30] Campistol JM, Abad C, Nogue S, Bertram A (1988). Acute renal failure in a patient treated by continuous povidone-iodine mediastinal irrigation. J Cardiovas Surg.

[31] Moriaski A, Hosono M, Murakami T, Sakaguchi M, Suehiro Y (2016). Effect of negative pressure wound therapy followed by tissue flaps for deep sternal wound infection after cardiovascular surgery: propensity score matching analysis. Interact Cardiovasc Thorac Surg.

[32] Debreneci T, Szerafin T, Galajda Z, Miskolczi S, Peterfly A (2008). Results of vauum-assisted wound closure system in the treatment of sternotomy wound infections following cardiac surgery. Magyar Sebeszet.

[33] Thurer RJ, Bognolo D, Vargas A, Isch JH, Kaiser GA (1974). The management of mediastinal infection following cardiac surgery. An experience utilising continuous irrigation with povidone-iodine. J Thorac Cardiovasc Surg.

[34] Wu S, Wan F, Gao YS, Zhang Z, Zhao H (2014). Sternal reconstruction of deep sternal wound infections following median sternotomy by single-stage muscle flaps transposition. Chinese medical science. Journal.

[35] Preminger BA, Yaghoobzadeh Y, Ascherman JA (2014). Management of sternal wounds by limited debridement and partial pectoralis major myocutaneous advancement flaps in 25 patients: a less invasive approach. Ann Plast Surg.

[36] Eriksson J, Huljebrant I, Nettelblad H, Svedjeholm R (2011). Functional impairement after treatment with pectoral muscle flaps because of deep sternal infection. Scand Cardiovasc J.

[37] Cabbabe EB, Cabbabe SW (2009). Surgical management of the symptomatic unstable sternum with pectoralis major muscle flaps. Plast Reconstr Surg.

[38] Tomos P, Lachanas E, Michail PO, Kostakis A (2006). Alternative bipectoral muscle flaps for postoperative sternotomy medistinitis. Ann Thorac Surg.

[39] Ascherman JA, Patel SM, Malhotra SM, Smith CR (2004). Management of sternal wounds with bilateral pectoralis major myocutaneous flaps in 114 consecutively treated patients: refinements in technique and outcome analysis. Plast Reconstr Surg.

[40] Sung K, Jun TG, Park PW, Park KH, Lee YT (2004). Management of deep sternal infection in infants and children with advanced pectoralis major muscle flaps. Ann Thorac Surg.

[41] Sung K, Niazi ZB, Salzberg CA, Permut LC (1998). Vascularised muscle flaps and reoperative approach for complicated, dehisced sternal wounds in children. Ann Plast Surg.

[42] Dosios T, Papadopoulos O, Mantas D, Georgiiou P, Asimacopoulos P (2003). Scandanavian journal of plastic and reconstructive surgery and. hand surgery.

[43] Backer CL, Pensler JM, Tobin GR, Mavroudis C (1994). Vascularised muscle flaps for life-threatening mediastinal wounds in children. Ann Thorac Surg.

[44] Ascherman JA, Hugo NE, Sultan MR, Patsis MC, Smith CR (1995). Single stage treatment of sternal wound complications in heart transplant recepients in whom pectoralis major myocutaneous advancement flaps were used. J Thorac Cardiovasc Surg.

[45] Li EN, Goldberg NH, Slezak S, Silverman RP (2004). Split pectoral major flaps for mediastinal wound coverage: a 12 year experience. Ann Plast Surg.

[46] Kuntscher MV, Mansouri S, Noack N, Hartmann B (2006). Verstality of vertical rectus abdominis musculocutaneous flaps. Microsurgery.

[47] Netscher DT, Eladoumikdachi F, Goodman CM (2001). Rectus abdominis muscle flaps used successfully for median sternotomy wounds after ipsilateral internal mammary artery ligation. Ann Plast Surg.

[48] Davison SP, Clemens MW, Armstrong D, Newton ED, Swartz W (2007). Sternotomy wounds. Plast Reconstr Surg.

[49] Lovich SF, Iverson LI, Young JN, Ennix CL, Harrell JE (1989). Omental pedicle grafting in the treatment of postcardiotomy sternotomy infection. Arch Surg.

[50] De Brabendere K, Jacobs TTD, Czapla J, La Meir M, Delavaux G (2012). Negative-pressure wound therapy and laparoscopic omentoplasty for deep sternal wound infections after median sternotomy. Tex Heart Inst J.

[51] Swartz WM, Luketich J, Quinlin RF, Edington H, Acarturk TO (2004). Laparoscopically harvested omental flap for chest wall and intrathoracic reconstruction. Ann Plast Surg.

[52] Salameh JR, Chock DA, Gonzalez JJ, Koneru S, Glass JL (2003). Laparoscopic harvest of omental flaps for reconstruction of complex mediastinal wounds. Journal of Society of Laparoendoscopic Surgeons.

[53] Schroeyers P, Wellens F, Degrieck I, De Geest R, Van Praet F (2001). Aggressive primary treatment for poststernotomy acute mediastinitis: our experience with omental and muscle flaps surgery. European Journal of Cardiothoracic surgery.

[54] Milano CA, Georgiade G, Muhlbaier LH, Smith PK, Wolfe WG (1999). Comparison of omental and pectoralis flaps for poststernotomy mediastinitis. Ann Thorac Surg.

[55] Parissis H, Al-Alao B, Soo A, Orr D, Young V (2011). Risk analysis and outcome of deep mediastinal wound infections with specific emphasis to omental transposition. J Cardiothorac Surg.

[56] Pieri M, Muller M, Scandroglio AM, Pergantis P, Kretschmar A (1992). Surgical treatment of mediastinitis with omentoplasty in ventricular assist device patients: report of referral centre experience. American Society for Artificial Internal Organs.

[57] Dornseifer U, Kleeberger C, Ehrl D, Herter F, Ninkovic M (2016). Arteriovenous loop-independent free flap reconstruction of sternal defects after cardiac surgery. J Reconstr Microsurg.

[58] Kaul P, Qadri SSA, Riaz M (2006). Chronic encapsulated mediastinal abscess presenting with remote cutaneous fistulisation 12 years after redo aortic valve replacement for prosthetic valve endocarditis. J Cardiothorac Surg.

[59] Telfer JR, Chapple DC, Powell BW (1996). Metastatic colonic adenocarcinoma in a pedicled omental flap used for sternal reconstruction: a case report. Br J Plast Surg.

[60] Meland NB, Arnold PG, Pairolero PC, Trastek VF (1990). Refinements in intrathoracic use of muscle flaps. Clin Plast Surg.

[61] Tizian C, Borst HG, Berger A (1985). Treatment of total sternal necrosis using the latissimus dorsi muscle flap. Plast Reconstr Surg.

[62] Hakaka T, Berg L, Berg E, Makinen K, Sipola P (2005). Repair of right ventricular free wall defect with a deepithelialized pedicled myocutaneous latissimus dorsi muscle flap. Ann Thorac Surg.

[63] Kaul P (2006). Repeated successful surgical rescues of early and delayed multiple ruptures of ventricular septum, right ventricle and aneurysmal left ventricle following massive biventricular infarction. J Cardiothorac Surg.

[64] Dejesus RA, Paletta JD, Dabb RW (2001). Reconstruction of the median sternotomy wound dehiscence using the latissimus dorsi myocutaneous flap. J Cardiovasc Surg.

[65] Taeger CD, Horch RE, Arkudas A, Schmitz M, Stubinger A (2016). Combined free flaps with arteriovenous loops for reconstruction of extensive thoracic defects after sternal osteomyelitis. Microsurgery.

[66] Wang J, Min P, Grasseti L, Lazzeri D, Zhang YX (2015). Preliminary outcomes of distal IMAP and SEAP flaps for the treatment of unstable keloids subject to recurrent inflammation and infections in the lower sternal and upper abdominal areas. J Reconstr Microsurg.

[67] Bravo FG, Schwarze HP (2009). Free style local perforator flaps: concept and classification system. Journal of Plastic, Reconstructive and Aesthetic Surgery.

[68] Cooper L, Payne C (2014). The use of novel inferomedial fasciocutaneous breast flaps to reconstruct a sternal dehiscence with concomitant soft tissue loss. Journal of Plastic, Reconstructive and Aesthetic Surgery.

[69] de Fontaine S, Devos S, Goldschmidt D (1996). Reduction mammoplasty combined with pectoralis major muscle flaps for median sternotomy wound closure. Br J Plast Surg.

[70] Pancholy B, Raman J (2015). Chest wall reconstruction using sternal plating in patients with complex sternal dehiscence. Ann Thorac Surg.

[71] Kalab M, Karkosa J, Kaminek M, Santavy P (2016). Transplantation of allogeneic bone graft in the therapy of massive post-sternotomy defects. Rozhl Chir.

[72] Lindsey JTA (2002). Retrospective analysis of 48 infected sternal wound closures: delayed closure decreases wound complications. Plast Reconstr Surg.

[73] Izzadoost S, Withers EH (2012). Sternal reconstruction with omental and pectoralis flaps:a review of 415 consecutive cases. Ann Plast Surg.

[74] van Wingerden JJ, Lapid O, Boonstra PW, de Mol BA (2011). Muscle flaps and omental flap in the management of deep sternal wound infection. Interact Cardiovasc Thorac Surg.

[75] Kaye AE, Kaye AJ, Pahk B, Mckenna ML, Low DW (2010). Sternal wound reconstruction : management in different cardiac populations. Ann Plast Surg.

[76] Spartalis E, Markakis C, Moris D, Lachanas E, Agathos E (2016). Results of the modified bipectoral muscle flap procedure for post-sternotomy deep wound infection. Surg Today.

